# Epitope Profiling of SARS-CoV-2 Spike Antigen Provides a Novel Strategy for Developing ELISAs Specific for Different Spike Protein Variants in Bivalent Vaccine Formulations

**DOI:** 10.3390/vaccines13080794

**Published:** 2025-07-26

**Authors:** Luciano Ettorre, Trevor Williams, Camille Houy, Shaolong Zhu, Michael Kishko, Ali Azizi, Andrew D. James, Beata Gajewska, Jason Szeto

**Affiliations:** 1 Analytical Sciences Immunology, Sanofi, 1755 Steeles Avenue West, Toronto, ON M2R 3T4, Canada; luciano.ettorre@sanofi.com (L.E.); trevor.williams@sanofi.com (T.W.); camille.houy2@sanofi.com (C.H.); ali.azizi@cepi.net (A.A.); 2 Analytical Sciences Biochemistry, Sanofi, 1755 Steeles Avenue West, Toronto, ON M2R 3T4, Canada; shaolong.zhu@sanofi.com; 3 Global Antigen Design (Virology), Sanofi, 200 West Street, Waltham, MA 02451, USA; michael.kishko@sanofi.com; 4 External Research and Development, Sanofi, 1755 Steeles Avenue West, Toronto, ON M2R 3T4, Canada; andrew.james@sanofi.com; 5 Analytical Sciences North America, Sanofi, 1755 Steeles Avenue West, Toronto, ON M2R 3T4, Canada; beata.gajewska@sanofi.com

**Keywords:** COVID-19, SARs-CoV-2, ELISA, antigenicity, potency, in vitro, antibody, biolayer interferometry, epitope, HDX-MS

## Abstract

Background/Objectives: An initial COVID-19 candidate vaccine containing a purified ancestral SARS-CoV-2 spike antigen was characterized with an ELISA using recombinant monoclonal antibodies (mAbs) generated against this variant. Upon the emergence of a new Beta (B.1.351) spike variant early in the pandemic, the assessment of a bivalent vaccine containing ancestral and Beta spike antigens began. Due to accelerated project timelines, mAbs generated specifically against the Beta spike antigen were not available at the time to address assay development and vaccine testing requirements. Methods: Using only the initial mAb panel raised against the ancestral spike antigen, an epitope-blocking ELISA strategy was developed to independently measure Beta spike antigen in bivalent vaccine formulations. To facilitate this, epitope profiling of spike antigens from both ancestral and Beta variants was performed with biolayer interferometry and hydrogen–deuterium exchange mass spectrometry using the original panel of mAbs. Results: The resulting blocking ELISA was precise and specific for the Beta spike antigen and detected the expected amount of this antigen in bivalent vaccine formulations. The specific amount of ancestral spike protein in the bivalent vaccine was also confirmed using the original ELISA developed at the onset of the pandemic. Conclusions: This epitope-blocking strategy helped to overcome key reagent availability issues and could be applied to other projects involving related proteins.

## 1. Introduction

The emergence of SARS-CoV-2, the causative agent of coronavirus disease 2019 (COVID-19), led to a pandemic that necessitated the rapid development of various vaccines to meet global demands. The SARS-CoV-2 spike glycoprotein (herein referred to as ‘spike antigen’) plays a crucial role for viral entry into host cells by binding to the angiotensin-converting enzyme 2 (ACE2) receptor, causing fusion of the viral envelope with the host cell membrane [1], further supporting its role as a critical immunodominant antigen and vaccine target [2]. Inhibiting this important step in the infection process is the main goal of current COVID-19 vaccines, making the spike antigen the key target antigen for neutralizing antibodies.

The native spike antigen forms a heavily glycosylated trimer in a pre-fusion conformation. Each native monomer is composed of S1 and S2 subunit domains [3]. The S1 receptor binding domain (RBD) mediates binding to the host cell ACE2 receptor, resulting in significant conformational changes: first to the S1 subunit domain, exposing proteolytic cleavage sites, which ultimately enables the S2 domain to mediate the insertion of its fusion peptide into the host cell membrane [4]. Subsequent hinge-like rearrangements act to pull the viral envelope and host cell membrane into close proximity, resulting in membrane fusion and transition of the spike antigen into its stable post-fusion conformation [4].

The ancestral spike antigen was replaced by a more dominant early variant ‘D614G’ spike antigen containing a crucial aspartic acid-to-glycine substitution that favors an open spike conformation, leading to higher affinity to the ACE2 receptor and increased viral infection and transmission [5,6]. These early variants became targets of intense vaccine development, including strategies involving mRNA technology, adenoviral vectors, and purified spike antigen [7,8]. Accompanying this was the need to rapidly develop suitable test methods to characterize, monitor, and/or release vaccine products. Typically, for vaccines containing purified proteins, such test methods include antibody-based assays (e.g., ELISAs) to detect and quantify the antigen. These assays can serve as in vitro potency assays, particularly if the selected antibodies can recognize immunologically relevant regions of the target antigen [9,10,11].

One major issue faced by antibody-based assays such as ELISAs is the generation of appropriate antibodies or ligands in a timely manner for assay development. Screening and acquiring monoclonal antibodies (mAbs) with traditional hybridoma methods is both costly and time-consuming. In the case of test method development for a protein-based COVID-19 vaccine, a hybridoma strategy to obtain mAbs would not have been optimal, given the demands of accelerated product development timelines during the COVID-19 pandemic. As such, for the Sanofi spike antigen-based COVID-19 vaccine, an alternative technology using phage display of human combinatorial antibody libraries (HuCAL) [12,13] was selected as a more rapid, animal-free, and cost-effective method to screen and produce human-derived ligand reagents for relevant potency immunoassay development.

Using a HuCAL strategy, we obtained and characterized several ligand candidates screened specifically with the ancestral SARS-CoV-2 spike antigen. Two of the ligands were subsequently selected to develop a quantitative sandwich ELISA to measure ancestral spike antigen in developmental vaccines, serving as an initial in vitro potency assay. Specifically, the ELISA measured the ‘antigenicity’ of the ancestral spike antigen vaccine candidate, a term describing the measure of epitope presentation and intactness, assessed through the binding of specific antibodies to an antigen [11].

As the pandemic progressed, several new variants of concern (VOCs) emerged, including the B.1.351 (Beta) lineage first identified in South Africa, which carried several consensus amino acid variations including three mutations in the spike antigen RBD (K417N, E484K, and N501Y). These mutations imparted conformational changes to the ACE2 binding region and decreased susceptibility to sero-neutralization [14]. The sensitivity of this strain to sera obtained from either convalescent non-vaccinated individuals who had recovered from COVID-19 infection or from individuals who had received a COVID-19 vaccine against the earlier strain was markedly reduced [15].

To meet the concern that newer variants could evade immune responses generated in vaccinated individuals, Sanofi initiated the assessment of a bivalent vaccine containing both ancestral and Beta spike antigens. In a Phase 3 double-blind placebo-controlled efficacy trial, a bivalent vaccine containing these two variants demonstrated efficacy against all circulating VOCs at the time, including the newer Omicron lineage [16]. The cross-protection against these VOCs suggests that vaccines containing the Beta spike antigen may be utilized as broad-coverage universal booster vaccines, rather than vaccines being constantly revised for newly emerging strains [17].

Due to highly accelerated project timelines to prepare for clinical trials, specific ligands for the Beta spike antigen were not available at the time to address the new testing demands for bivalent vaccines containing two unique, but highly related, SARS-CoV-2 spike antigens. HuCAL screening of new ligands specific for the Beta variant was initiated; however, this would still require upwards of several months for new ligand characterization, selection, and scale-up of selected candidates for assay development and implementation.

We describe here the development of a quantitative antigenicity ELISA involving an epitope-blocking strategy that served to specifically measure Beta spike antigen in bivalent vaccine formulations using only the original HuCAL antibody panel raised against the ancestral variant. This was achieved through epitope profiling with this mAb panel on both ancestral and Beta spike antigen variants using biolayer interferometry (BLI) and hydrogen–deuterium exchange mass spectrometry (HDX-MS). This led to the identification of an appropriate blocking antibody that preferentially bound to the ancestral spike antigen in bivalent vaccine formulations, to prevent its undesired detection by a subsequent antibody which served to specifically detect only the Beta variant in the sample. This interim solution was used to ensure the continuity of vaccine development, until ligands specific for the Beta spike antigen could be delivered from the HuCAL platform. In addition, the initial ELISA developed to detect the ancestral spike antigen in the monovalent vaccine was shown to remain specific for the ancestral antigen, even in bivalent formulations.

## 2. Materials and Methods

### 2.1. Generation of the SARS-CoV-2 Recombinant Prefusion Spike Antigen

Recombinant ancestral and Beta SARS-CoV-2 prefusion spike antigens were produced from a Sanofi proprietary cell culture technology using an insect cell baculovirus expression vector system, as outlined previously [18,19]. Sequence alignment for the ancestral and Beta spike antigens is presented in Supplementary Figure S1. Information on the spike constructs and specific domains has been published previously [19].

### 2.2. HuCAL Screening and Generation of Ligands Specific to Ancestral SARS-CoV-2 Spike Antigen

Ligands recognizing the S1 and S2 domains of the ancestral SARS-CoV-2 spike antigen were generated using HuCAL technology (BioRad, Neuried, Germany). For the S1 specific ligands, the HuCAL PLATINUM phage library was first panned for clones recognizing full-length the ancestral prefusion spike antigen (produced by Sanofi, Toronto, ON, Canada) and the receptor binding domain (RBD) from the early pandemic Wuhan-Hu-1 spike antigen variant (Cat# 40592-V08B) (Sino Biological, Pennsylvania, PA, USA). Counterselection was performed against the SARS-CoV-1 spike antigen (Cat# 40634-V08B) (Sino Biological, Pennsylvania, PA, USA) for the depletion of unspecific binders.

For S2-specific ligands, the HuCal PLATINUM phage library was first panned for clones recognizing the full-length ancestral prefusion spike antigen (produced by Sanofi) and the S2 subunit of SARS-CoV-2 from the early-pandemic Wuhan-Hu-1 variant (Cat# 40590-V08B) (Sino Biological, Pennsylvania, PA, USA). Counterselection was performed against the SARS-CoV-1 spike antigen for the depletion of unspecific antibodies. After the panning, the enriched antibody gene pool from the phage display vector was subcloned into a bacterial (*Escherichia coli*) expression vector, where the ligands would be expressed in a final fragment antigen-binding (Fab) antibody format. After the pre-screening of clones using ELISA, fifteen of the monovalent human Fab antibodies were covalently coupled to the human IgG Fc region using the SpyTag-SpyCatcher technology (BioRad, Germany).

### 2.3. Pseudovirus-Based Neutralization Assays

The reporter virus particle (RVP)-GFP containing spike antigen from the SARS-CoV-2 Wuhan Hu-1 strain, isolate WIV04 (Wuhan, China; Genbank accession MN996528.1), was obtained from Integral Molecular (Cat# RVP-701G) (Integral Molecular, Pennsylvania, PA, USA). Candidate mAbs were diluted to an initial concentration of 1.25 mg/mL and 12.5 µg/mL in media (FluoroBrite™ phenol-red-free DMEM  +  10% FBS  +  10 mM HEPES  +  1% PS  +  1% GlutaMAX™) (ThermoFisher, Waltham, MA, USA). Further, a two-fold, 11-point dilution series was performed in media for each initial dilution. Diluted mAbs were mixed with RVP-GFP diluted to contain ~300 infectious particles per well and incubated for 1 hour at 37 °C. Ninety-six-well plates of ~50% confluent 293T-hsACE2 clonal cells (Cat# C-HA102) (Integral Molecular, Pennsylvania, PA, USA) in 75  µL volume were inoculated with 50  µL of the mAb and virus mixtures and incubated at 37  °C for 72  h. At the end of the incubation, plates were scanned on a high-content imager and individual GFP-expressing cells were counted. The inhibitory concentration titer (IC50) was reported as the mAb concentration that reduced the number of virus plaques in the test by 50%.

### 2.4. Biolayer Interferometry (BLI) Equipment and General Reagents for BLI Experiments

Biolayer interferometry (BLI) was conducted on an Octet 384 System (Sartorius, Göttingen, Germany). BLI assays were conducted with mAbs and samples diluted in 1X Kinetics Buffer (1X KB) (Sartorius, Germany). Where required, mAbs or other protein biotinylation was performed using EZ-Link NHS-PEG4-Biotin kit with Zeba Spin Desalting Columns (7K molecular weight cut-off) as per manufacturer’s instructions (Thermo Scientific, Waltham, Massachusetts, MA, USA). Capture of biotinylated mAbs or proteins was performed using streptavidin biosensors (Sartorius, Germany).

### 2.5. Assessment of mAbs for ACE2 Receptor Binding Inhibition Activity Using Biolayer Interferometry (BLI)

Purified spike antigen was incubated with varying concentrations of each mAb in 1X KB. The final concentration used was 20 µg/mL of ancestral or Beta spike antigen incubated in antibody concentrations of 100 µg/mL, 25 µg/mL, 6.25 µg/mL, and zero antibody at room temperature (22 °C) for 90 min.

Purified ACE2 receptor (Syd Labs, Hopkinton, MA, USA) was biotinylated and loaded onto streptavidin biosensors at 20 µg/mL in an Octet 384 system, followed by a washing step in 1X KB. Biosensors were subsequently exposed to the mAb and spike antigen mixtures for a 180 s association step, followed by a 180 s dissociation step in 1X KB. A qualitative ranking of the ability of each mAb to inhibit spike antigen binding to the ACE2 receptor was obtained by comparing magnitude binding signals of the spike antigen (pre-incubated with or without various amounts of mAb) to the ACE2 receptor.

### 2.6. Antibody Binding Studies on Ancestral and Beta Spike Antigens Using BLI

The ancestral and Beta spike antigen variants were thermally stressed at either 37 °C, 45 °C, or 60 °C for 4 days. These samples, along with an untreated spike antigen stored at 2–8 °C, were analyzed for their binding to each mAb. Biotinylated mAbs were loaded onto streptavidin biosensors at 20 µg/mL (300 s) followed by a 60 s washing step in 1X KB to establish a baseline. The biosensors were then exposed to the thermally stressed ancestral or Beta spike antigen at 20 µg/mL in 1X KB buffer for a 300 s association step, followed by a final 300 s dissociation step in 1X KB buffer. The mAbs were assessed qualitatively and ranked by their ability to exhibit a reduced signal when binding to the thermally stressed spike antigen compared to unstressed protein.

For measuring binding affinity, mAbs were loaded onto biosensors as described above and subsequently exposed to the spike antigen at seven concentration levels ranging from 1.6 nM to 100 nM for 500 s, with a dissociation step performed for 1100 s. A negative control (biosensor with no mAb) was used to subtract any background signal from non-specific binding of the protein to the biosensor. The kinetic data was obtained by applying a global fit (1:1 model) to the binding response.

In instances where rapid qualitative screenings of the relative binding strength of mAbs to the spike antigen were needed, only one antigen concentration level (40 nM) and shorter intervals for antibody–protein association (300 s) and dissociation (600 s) steps were used.

### 2.7. ELISA for Ancestral SARs-CoV-2 Spike Antigen

ELISA microtiter plates were coated with the capture monoclonal antibody clone 875 (mAb 875) specific to the S2 domain of the SARS-CoV-2 spike antigen. A representative purified in-house ancestral spike antigen batch was used as a reference standard. Following sample and reference standard dilutions, capture incubation, and washes, antigen detection was performed by the addition of a mAb specific to the S1 domain of the ancestral spike antigen (mAb 886) conjugated with horseradish peroxidase (HRP). Following incubation and washing, a substrate solution (tetramethylbenzidine (TMB)) was added, and the HRP-catalyzed colorimetric reaction was stopped by the addition of 2 N sulfuric acid. ELISA plates were read by measuring absorbance at 450 nm wavelength with a reference wavelength of 540 nm. The ELISA data was analyzed with SoftMax Pro version 6.5.1 GxP software (Molecular Devices, San Jose, CA, USA) using a 4-PL model. Parallelism was assessed using the equivalence test approach of the slope ratio, whereby the confidence intervals of the test article must be within set equivalence limits to prove parallelism with the respective reference standard. The relative potency was determined and an antigenicity value for the sample reported as ‘ELISA test units’ per mL (ETU/mL). This ELISA was used to measure the ancestral spike antigen in bivalent formulations either with a monovalent or bivalent reference standard.

### 2.8. Epitope Mapping by Hydrogen–Deuterium Exchange Mass Spectrometry (HDX-MS)

Epitope screening for certain mAbs was conducted by HDX-MS. The non-deuterated control buffer used for digestion control and the buffer for deuteration were described previously [19]. Antigen and antibody complexes were formed by incubating at an equimolar concentration of 1.1 µM at room temperature for an hour. For epitope screening, two time points (10 and 30 min) were acquired in triplicate. The quenched samples were injected into a NanoAcquity UPLC HDX module housing a 1:1 pepsin/protease XIII enzyme column (NovaBioAssays, Woburn, MA, USA) for digestion. The resulting peptides were desalted (100 µL/min for 3 min) with a Waters BEH C18 guard column and separated using an ACQUITY CSH C18 analytical column (30 µL/min using 7 min gradient). The ionKey (Waters Corp., Milford, MA, USA) was used as an electrospray source and the eluted peptides were detected using a Waters Synapt G2-Si mass spectrometer (Waters Corp., MA, USA) acquiring a range from 300 to 1700 *m*/*z*. GluFib (785.8426 *m*/*z*) was used as a lock mass solution to maintain a mass calibration of <10 ppm. ProteinLynx Global Server (Waters Corp., MA, USA) was used for peptide identification while DynamX software (version 3.0.0; Waters Corp., MA, USA) was used for the analysis of deuterium exchange. Quantitative HDX data analysis to identify potential epitopes was performed as previously described [20]. The final epitope sequences were determined by averaging overlapping peptide data rather than evaluating individual peptide data. This was performed to optimize and refine the epitope length. Peptide averaging was performed using the “heatmap” view feature of the DynamX software, which relies on relative fractional uptake (RFU). This normalizes the differences in peptide lengths and the exchangeable amides (the first amino acid is always excluded since it is a rapid back-exchanger). The resulting differences were mapped onto representative 3D structures of the ancestral variant, PDB: 6XM3, and Beta variant, PDB: 7LYQ, using Schrödinger, LLC. (2010) the PyMOL Molecular Graphics System, Version 2.4.0 (New York, NY, USA).

### 2.9. Using Biolayer Interferometry (BLI) to Establish Proof of Concept of Blocking ELISA

Biotinylated mAb clone 875 was first immobilized onto streptavidin biosensors at 20 µg/mL, followed by a washing step in 1X KB. Sensors were then exposed to either the ancestral or Beta spike antigen (20 µg/mL) for 300 s to capture each antigen, followed by a 60 s wash in 1X KB. Biosensors were then immersed in a solution containing the candidate blocking mAb (20 µg/mL) for 300 s, followed again by a 300 s wash in 1X KB. Lastly, biosensors were exposed to a mAb candidate (20 µg/mL) that would serve as the detection mAb for the Beta spike antigen in the final blocking ELISA.

### 2.10. Epitope-Blocking ELISA for Specific Detection of SARS-CoV-2 Beta Spike Antigen Variant

ELISA microtiter plates were coated with mAb 875, which recognizes the S2 domain of both the ancestral and Beta spike antigens. The reference standards used consisted of either a monovalent in-house Beta spike antigen or a bivalent formulation consisting of equal amounts of in-house ancestral and Beta spike antigens. Following sample incubation and plate washing, an epitope-blocking step was performed by incubation with the anti-S1 domain mAb 879. This blocking mAb inhibits the binding of the final detection antibody on the ancestral spike antigen, but not on the Beta variant. After epitope-blocking and a washing step, the final HRP-conjugated detection antibody (mAb 876, recognizing the anti-S1 domain) was added for the detection of Beta spike antigen specifically. Following incubation and washing, the ELISA plate was processed and analyzed as described above.

### 2.11. Qualification of ELISAs

The epitope-blocking ELISA for the Beta spike antigen and the original ELISA for the ancestral spike antigen were qualified by assessing the parameters of accuracy, precision (repeatability and intermediate precision), specificity, and linearity. The assays were qualified using five different bivalent spike antigen formulations containing from 12.5% to 200% of each nominal spike antigen concentration. The samples were tested by two analysts over three days. Accuracy was assessed in terms of the percent recovery of the specific antigenicity (either Beta or ancestral) compared to the respective expected antigen concentrations. Intermediate precision and linearity across all levels were assessed. The monovalent Beta spike antigen reference standard was used in the assay qualifications for the epitope-blocking ELISA, and the monovalent ancestral spike antigen reference standard was used for the ancestral spike antigen ELISA.

## 3. Results

### 3.1. Characterization of Antibodies Generated Against Ancestral SARS-CoV-2 Spike Antigen

HuCAL technology was employed shortly after the onset of the pandemic to screen for ligands targeting the original ancestral SARS-CoV-2 spike antigen vaccine candidate. A panel of 15 candidates (13 binding to the S1 domain, and 2 binding to the S2 domain) were identified, which were further characterized to select those suitable for developing a sandwich-style antigenicity ELISA to use as an in vitro vaccine potency test. Desirable characteristics included mAbs with a higher affinity, neutralizing ability, binding to distinct spike antigen sites (important for sandwich-style ELISAs), and ability to detect alterations to the target protein (e.g., disruption of epitopes in heat-stressed protein).

The characterization data for a subset of the candidate antibodies of interest are shown in Table 1 below. Characterization data for all 15 candidate antibodies are presented in Supplementary Table S1. Rapid initial screening indicated mAb clone 886 had one of the highest affinities amongst the thirteen S1 domain-specific binders, while mAb 875 had the highest affinity of the two S2 domain-specific binders (Table 1 and Supplementary Table S1).

Using a pseudovirus neutralization assay, several of the anti-S1 domain mAbs, including clones 876, 879, 886, and 962, demonstrated effective neutralization, with IC50 values less than 100 ng/mL (Table 1 and Supplementary Table S1).

In general, the majority of mAbs that presented a neutralizing capability could also reduce the interaction of purified spike antigen with the ACE2 receptor, including clones 876, 879, 886, and 962 (see Table 1, Supplementary Table S1, and Supplementary Figure S2A,B for example BLI response curves). Furthermore, all the mAbs detected a native spike antigen and did not bind to denatured samples (see Supplementary Figure S3 for denaturation procedure and data), indicating they likely recognized conformational epitopes.

**Figure 1:**
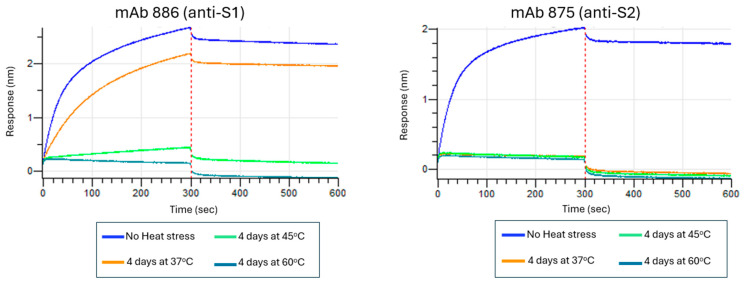
Assessing the ability of anti-spike protein monoclonal antibodies to detect the disruption or loss of epitope integrity in a heat-stressed ancestral spike antigen using BLI. Sensors captured each mAb and were subsequently incubated with various heat-stressed spike protein samples (previously treated for 4 days at 37 °C, 45 °C, and 60 °C) as indicated.

The ability of the mAbs to detect the disruption or loss of epitope integrity in a heat stress-induced antigen was also examined with BLI. Increasing heat stress on spike antigen resulted in lower BLI binding signals, indicative of a loss of spike protein epitope integrity for each antibody, further supporting that the mAbs recognized conformational epitopes. Figure 1 shows examples of this BLI screening performed on mAbs 886 (anti-S1 domain mAb) and 875 (anti-S2 domain mAb).

The mAbs 886 and 875 were ultimately selected as the preferred candidates for the development of an antigenicity ELISA for the ancestral SARS-CoV-2 spike antigen. Both mAbs bound with high affinity to conformational epitopes in separate domains of the target protein (mAb 886 binding to the S1 domain containing the RBD, and mAb 875 binding to the S2 domain). Furthermore, the anti-RBD mAb 886 had a neutralizing function and the ability to inhibit the spike antigen’s interaction with the purified ACE2 receptor, and both mAbs selected for the sandwich ELISA were sensitive to heat-induced disruptions in their epitopes.

### 3.2. Antigenicity ELISA Development for Ancestral SARS-CoV-2 Spike Antigen

A sandwich ELISA strategy with mAb 875 (anti-S2 domain) as the capture antibody and mAb 886 (anti-S1 RBD) conjugated to horseradish peroxidase (HRP) as the detection antibody was used. From the assay optimizations, the 16-point four-parameter logistic (4-PL) curves exhibited clear upper and lower asymptotes (Supplementary Figure S4). Both the purified ancestral spike protein reference standard and a similarly purified sample lot showed parallelism (Supplementary Figure S4).

**Figure 2:**
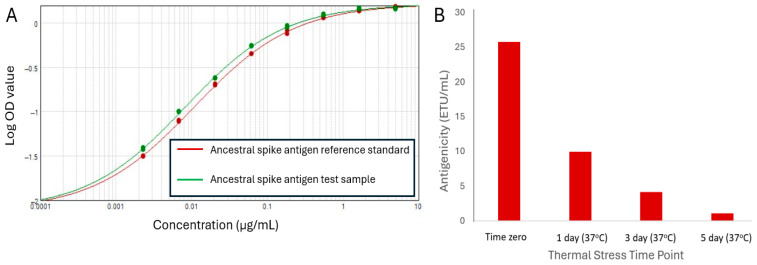
(**A**) Example of 4-PL response curves for the ancestral spike antigen antigenicity ELISA using 8-point dilutions and anchoring of curves to the median optical density value of blank ELISA wells. (**B**) The ancestral spike antigen antigenicity ELISA shows decreasing detection of the heat-stressed spike antigen with increased duration of heat treatment. Results from a single experiment are shown.

As the lowest-concentration reference standard points were consistently at background signal levels, the response curve was anchored to the median optical density (OD) value of the blank ELISA wells and simplified to an eight-point reference standard with 3-fold serial dilutions (Figure 2A).

The ability of the sandwich ELISA to serve as a stability-indicating assay was assessed using the heat-stressed spike antigen treated at 37 °C for up to 5 days. A clear drop in antigenicity values (reported as ELISA test units per mL; ETU/mL) over the course of the study was observed (Figure 2B), confirming that the antigenicity ELISA for the ancestral spike antigen was stability-indicating.

### 3.3. Developing an Epitope-Blocking ELISA Strategy to Detect Beta Spike Antigen in Bivalent Vaccine Formulation

Progression of the COVID-19 pandemic and rapid emergence of new variants of concern led to the consideration of bivalent vaccine formulations containing spike antigens from two different SARS-CoV-2 strains at the time, namely ancestral and Beta variants (see Supplementary Figure S1 for the sequence alignments of these variants). However, despite the relatively rapid HuCAL screening process, suitable antibodies specific for the Beta variant would not be available in a timely manner to address accelerated project testing demands for the bivalent vaccine formulation.

**Figure 3:**
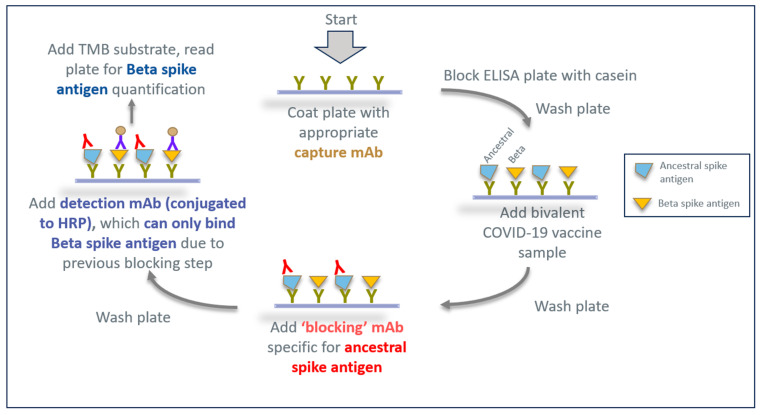
Overview of the epitope-blocking ELISA for the specific detection of the Beta spike antigen in bivalent vaccine formulations containing both ancestral and Beta spike antigens. The ELISA plate is coated with a capture antibody that can bind to both the ancestral and Beta spike antigens. After blocking the ELISA plate with casein and washing it, a bivalent vaccine sample is added. After incubation, the plate is washed and a suitable anti-S1 domain mAb is added to specifically bind to the ancestral spike antigen target to block its subsequent detection in the ELISA procedure. The plate is washed and a different final detection mAb (conjugated to HRP) is added, binding only to the Beta spike antigen due to previous epitope blocking on the ancestral variant.

Since the only available mAb reagents were generated against the ancestral spike antigen, we investigated a strategy using the initial panel of HuCAL antibodies to identify an anti-S1 domain mAb that could block the detection of the ancestral antigen and yet allow the subsequent detection of the related Beta spike antigen S1 domain by another mAb. A schematic of this ‘epitope-blocking ELISA’ strategy for monitoring Beta spike protein antigenicity in a bivalent vaccine is shown in Figure 3. Here, a blocking step using an existing mAb from the original mAb panel (generated against the ancestral RBD) would bind to the ancestral spike antigen, forcing a final detection antibody (conjugated to HRP) to bind solely to the Beta variant antigen in the same bivalent sample.

**Figure 4:**
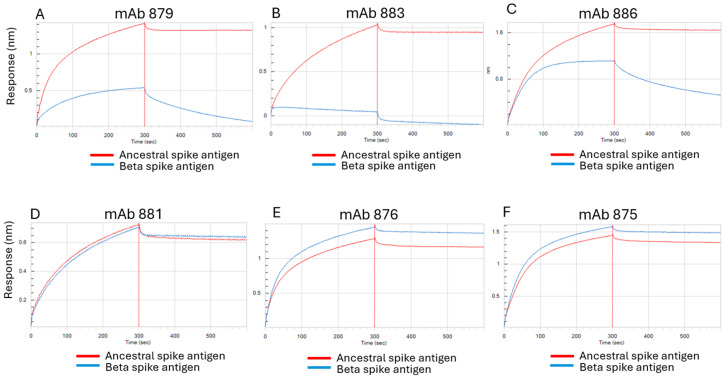
BLI assessment of various monoclonal antibodies binding to the ancestral and Beta spike antigens. (**A**–**C**) Anti-S1 domain mAbs 879, 883, and 886 exhibited preferential binding to the ancestral spike antigen. (**D**,**E**) Examples of anti-S1 domain mAbs exhibiting generally similar binding to both spike antigen variants. (**F**) The anti-S2 domain mAb 875 exhibited generally similar binding to both variants.

The original anti-S1 mAb panel was thus screened to identify mAbs with the appropriate qualities and epitopes needed to fulfill the critical “blocking” (red font, Figure 3) and “detection” roles (blue font, Figure 3). First, BLI was used to identify three mAb clones (clones 879, 883, and 886) that exhibited preferential binding to the ancestral spike antigen and not to the related Beta variant (Figure 4A–C). Both mAbs 879 and 886 exhibited some initial binding to the Beta variant; however, this binding was very weak, as evidenced by the rapid dissociation from this antigen after the initial interaction (Figure 4A,C, blue sensorgrams). Many of the remaining anti-S1 domain mAbs exhibited generally similar binding to both spike antigens (see Figure 4D,E for typical examples). BLI also confirmed that the anti-S2 domain mAb 875 (used as the capture antibody in the initially developed ancestral spike antigen antigenicity ELISA) was able to bind well to both the ancestral and Beta spike antigens (Figure 4F); hence, it could serve as a suitable ELISA capture antibody for both variants due to a conserved epitope(s).

From the three candidate blocking mAbs, clone 883 was not selected for further development as it had lower affinity for the ancestral spike antigen and lower pseudovirus neutralization (Supplemental Table S1). Full binding kinetics analyses confirmed both mAb 879 and mAb 886 had stable high-affinity binding to the ancestral spike antigen and weak affinity for the Beta variant, particularly with mAb 879 (Supplementary Table S2 and Figure 4A,C), thus making this mAb clone the more ideal candidate blocking antibody. Furthermore, as mAb 886 was already used as the detection antibody in the previously developed ELISA for the ancestral spike antigen (Figure 2), mAb 879 was ultimately selected as the blocking mAb.

Next, we sought to identify a mAb to use for detection of the Beta antigen, after the blocking step is applied to the ancestral spike antigen in the ELISA (Figure 3). The ideal candidate mAb should bind well to the Beta spike antigen and be effectively competed with by mAb 879 on the ancestral spike antigen. The mAbs 883 and 886 had already been shown to have low-affinity binding to the Beta variant (Figure 4B,C), making them less-than-ideal candidates for the detection of this variant.

**Figure 5:**
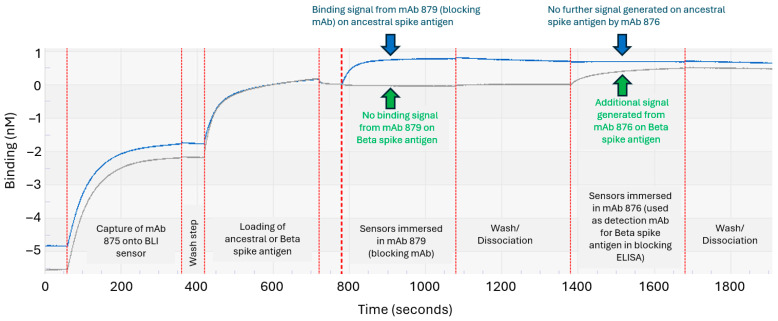
Using BLI to confirm the ability of mAb 879 to block the detection of the ancestral spike antigen by mAb 876. The mAb 875 (used as a spike antigen capture antibody in the blocking ELISA) was first immobilized on BLI biosensors. Sensors were then exposed to ancestral or Beta spike antigen to allow binding. The sensors were then placed into a solution of the blocking mAb candidate (mAb 879), washed, and then exposed mAb 876 (the candidate detection mAb for the Beta spike antigen in the blocking ELISA). The sensors that were originally loaded with the ancestral spike antigen (sensorgram marked by blue arrows) did not generate any further signals upon exposure to mAb 876 (rightmost blue arrow), indicating that the mAb 876 epitope was no longer available to bind. The sensors loaded with the Beta spike antigen (sensorgram marked by green arrows) were still able to generate a signal upon exposure to mAb 876, indicating its epitope on this variant was not blocked.

The remaining candidate anti-S1 mAbs were further assessed for their suitability as detection mAbs for Beta spike antigen in the blocking ELISA. Again, BLI proved its usefulness in decision making by allowing epitope binning-style assessments to simulate key steps in the blocking ELISA and the real-time visualization of mAb performance. An example is shown in Figure 5, where the capture mAb 875 was first immobilized on BLI biosensors and subsequently bound to the purified ancestral or Beta spike antigen. The sensors were then immersed in a solution containing the blocking mAb 879, followed by a washing step. Lastly, the sensors were immersed into solutions of one of the six remaining candidate detection mAbs (mAb 876 is shown as an example in Figure 5, and mAb 962 is shown as another example in Supplementary Figure S5). Sensors that were immersed in mAb 876 did not generate any further BLI signals for the ancestral spike antigen (rightmost blue arrow, Figure 5), while a further BLI signal was observed for the Beta variant (rightmost green arrow, Figure 5). The results indicated that the mAb 876 epitope on the ancestral spike antigen was fully blocked by mAb 879, a key step in the proposed epitope-blocking ELISA. In contrast, sensors immersed in mAb 962 could still generate a signal on the ancestral spike antigen, indicating that the mAb 962 epitope was not fully blocked (Supplementary Figure S5, orange arrow); hence, this example showed a mAb that was not suitable for use as a detection mAb for the Beta variant in bivalent formulations. These results also confirmed that mAb 879 was acting as a suitable ‘blocking’ mAb for the ancestral spike antigen, a key step of the proposed epitope-blocking ELISA for testing bivalent formulations (Figure 3).

Additional functional data further supported the selection of mAb 876 as a suitable candidate for detecting Beta spike antigen in the blocking ELISA. Notably, this mAb had high affinity for the Beta spike antigen (Supplementary Table S2), was able to reduce the binding of this antigen to the ACE2 receptor in a BLI assay (Supplementary Figure S2C) and had a stability-indicating capacity due to its reduced binding to the heat-stressed Beta spike antigen (Supplementary Figure S6).

### 3.4. Assessing the Epitope-Blocking ELISA’s Ability to Detect Beta Spike Antigen in Bivalent Vaccine

Following experiments to optimize the concentration of the blocking mAb 879 used in the epitope-blocking ELISA, bivalent formulations containing varying amounts of both the ancestral and Beta spike antigens were used to assess the assay specificity. Two different types of reference standards were used during assay development, consisting of either a monovalent purified in-house Beta spike antigen or a bivalent formulation containing both variants. Supplementary Figure S7 shows an example of eight-point 4-PL ELISA response curves of the monovalent Beta spike antigen reference standard and a bivalent sample, with anchoring of the curves to the median optical density (OD) value of the ELISA blank wells.

The results showed that the Beta spike antigenicity values measured using the epitope-blocking ELISA aligned well with the expected recoveries (between 101% and 119% recovery) in each of the bivalent formulations tested (Supplementary Table S3). Importantly, the results confirmed the specificity of the blocking ELISA for the Beta spike antigen, as no measured result was obtained in a sample containing only the ancestral spike variant. Moreover, there was overall low assay variability (n = 3 with a coefficient of variation (%CV) less than 10%) for each formulation tested by two analysts over three days (Supplementary Table S3; results were obtained using the bivalent formulation as a reference standard in this example).

Further studies using the monovalent Beta reference standard confirmed excellent recoveries of the Beta spike antigen in various bivalent formulations when tested using the blocking ELISA, with recoveries between 96% and 102% (Table 2, green columns). The results further confirmed the specificity of the blocking ELISA for the Beta spike antigen, as no measured result was obtained in a sample containing only the ancestral variant (Table 2).

When the bivalent samples were tested with the blocking ELISA but without any ‘blocking step’ (i.e., without mAb 879), the reported ELISA values for all bivalent samples were higher in all cases (ranging from ~1.2 to 1.4-fold higher) compared to the values obtained when the blocking step was applied (Table 2, orange column vs. green columns). This was not unexpected, as the absence of the critical ‘blocking step’ leads to an additional ELISA signal arising from the binding of the ancestral spike antigen by the detection mAb 876.

The bivalent formulations were also tested with the initial ELISA originally developed for the monovalent ancestral spike antigen vaccine (using mAb 875 for capture and mAb 886 for detection). This ELISA provided good recoveries of the ancestral spike antigen in comparison to expected formulation levels (Table 2, gray columns). The specificity of this ELISA was also confirmed, as no measured result was obtained in a sample containing only the Beta spike antigen. The specificity of the original ELISA was imparted by mAb 886, as this antibody showed high affinity for the ancestral spike antigen, and only weak binding with a rapid dissociation from the Beta variant (Figure 4C and Supplementary Table S2).

Overall, the results show that the epitope-blocking ELISA and the original ancestral spike antigen ELISA can be used to specifically measure Beta and ancestral spike protein antigenicity, respectively, in bivalent formulations. Due to the specificity of the blocking ELISA, the data also supports the notion that either monovalent Beta or bivalent formulations could serve as reference standards for this assay when testing bivalent formulation samples. In addition, the data supports the notion that mAb 875 (anti-S2 domain) was a suitable capture mAb for both Beta and ancestral spike antigens in the different ELISAs, as supported by previous BLI data (Figure 4F).

### 3.5. Epitope Mapping of mAbs Using HDX-MS

Hydrogen–deuterium exchange by mass spectrometry (HDX-MS) was used to map the epitope regions of the mAbs used in the two ELISAs to gain more insight into how they contribute to their roles in their respective assays. The results are summarized in Table 3.

**Figure 6:**
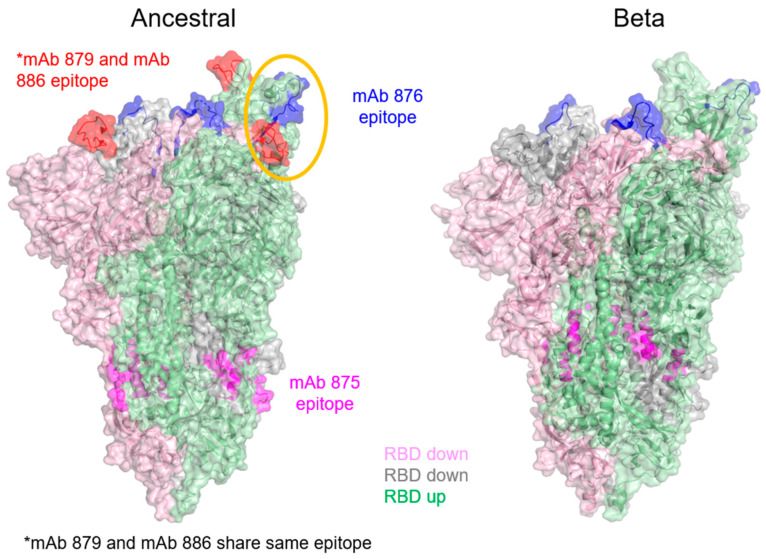
Epitopes for mAbs 876, 879, 886, and 875 mapped onto representative three-dimensional trimeric spike antigen structures of the Beta (PDB: 7LYQ) and ancestral (PDB: 6XM3) variants. Epitopes for mAb 876, 879, and 875 are highlighted in blue, red, and magenta, respectively. Note that mAb 886 shares the same epitope as mAb 879; hence, they are grouped together in red. Steric hindrance resulting in close proximity of mAb 876 and mAb 879 epitopes are shown in the orange ellipse. Two spike antigen protomers with RBD in the ‘down’ configuration are shown in pink and gray, whereas a third protomer with RBD in the ‘up’ configuration is shown in green.

The epitope for mAb 879 comprises residues 459–475 for the ancestral spike antigen, confirming that the blocking mAb bound to the RBD. HDX-MS did not detect any notable binding of this mAb to the Beta variant, as expected (Table 3 and Supplementary Figure S8). The mAb 876 (used as the detection antibody in the blocking ELISA) had epitope regions identified on both ancestral and Beta spike antigens highlighting a common overlapping sequence (Table 3 and Supplementary Figure S8). The epitope region for this detection mAb was also in close proximity (within 20 amino acids) to the epitope region of the blocking mAb 879, found only on the ancestral spike antigen (Table 3). As such, the binding of the blocking mAb 879 to the ancestral spike antigen could result in steric hindrance that interferes with the subsequent binding of the ELISA detection mAb 876 to this spike antigen variant (Figure 6, yellow circled region shown on one protomer in the ancestral spike antigen trimer). Note that mAb 886, which was used as the detection mAb for the specific ELISA against the ancestral spike antigen, shared the same epitope as mAb 879 (Table 3 and Figure 6) and was confirmed to bind poorly to the Beta variant (Figure 4C).

The epitope region for mAb 875, used as the capture antibody for both ancestral and Beta spike antigen ELISAs, was also determined. As expected, this anti-S2 mAb showed a common binding site between the ancestral and Beta antigens (Table 3, Figure 6, and Supplementary Figure S9), further supporting its suitability to act as a general ELISA capture mAb for both variants’ antigens. There was no sequence coverage at residues 771–781 in the raw data for the Beta spike antigen due to inadequate HDX-MS spectral quality.

### 3.6. Assessing Stability-Indicating Ability of the Epitope-the Blocking ELISA

**Figure 7:**
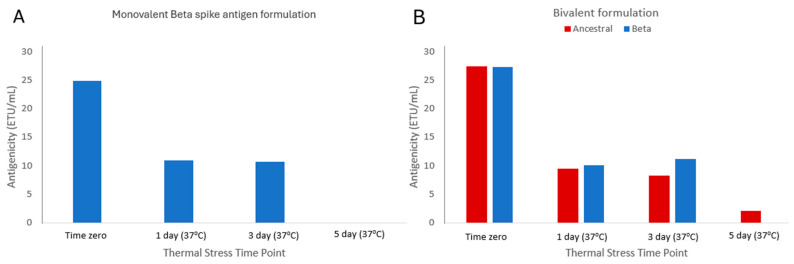
Assessment of spike antigen antigenicity in heat-stressed (**A**) monovalent Beta spike antigen formulations and (**B**) bivalent spike antigen formulations. Blue bars indicate results for Beta spike antigen and red bars indicate results for ancestral spike antigen, obtained with their respective specific ELISAs. Results from single experiments shown.

Having confirmed the assay is specific for Beta spike antigen in bivalent formulations, the ability of the epitope-blocking ELISA to detect a disruption or loss of binding to the heat-stressed spike protein was assessed using heat-treated mono- and bivalent developmental vaccine formulations incubated at 37 °C for up to 5 days. Note, the monovalent heat-stressed Beta spike antigen samples were tested without the use of the blocking mAb 879 since there was no ancestral spike variant present in those formulations. Figure 7A,B (blue bars) show that the Beta spike antigenicity values decreased over the course of the study in both mono- and bivalent samples, respectively, confirming that the ELISA was stability-indicating for this antigen. In addition, the original ELISA (specific for the ancestral spike antigen) was also able to detect a drop in antigenicity in the heat-treated bivalent samples over time (Figure 7B, red bars).

### 3.7. Qualification of ELISAs

The epitope-blocking ELISA for the Beta spike antigen and the original ELISA for the ancestral spike antigen were qualified by assessing the parameters of accuracy (% recovery), intermediate precision, specificity, and linearity. Supplementary Table S4 summarizes the various bivalent samples used in assay qualification. The blocking ELISA qualification data presented here (Table 4) were generated using monovalent Beta spike antigen reference standard to test the various formulations. Since the bivalent reference standard was also used to test the bivalent samples and gave similar results, only the data generated with the monovalent Beta spike antigen reference standard is presented below.

The results from the blocking ELISA qualification showed %CVs of less than 10% for all levels tested (n = 3 data points for each level, obtained by two analysts) and average % recoveries ranging from 96% to 103%.

The linearity of the epitope-blocking ELISA was confirmed by performing a linear regression analysis for the observed averages of log antigenicity (ETU/mL) vs. log expected concentrations (µg/mL) showing an R^2^ value greater than 0.99 (Supplementary Figure S10). The specificity of the ELISA for the Beta spike antigen in bivalent formulations was previously confirmed, as described in the above sections.

Assay qualification for the original ELISA developed for the ancestral spike antigen also showed similar results for accuracy, intermediate precision, and linearity on the various bivalent samples (Supplementary Table S5 and Supplementary Figure S11). Overall, these results demonstrated the suitable performance of the epitope-blocking ELISA for measuring Beta spike antigenicity and of the original ELISA for measuring ancestral spike antigenicity in bivalent formulations.

## 4. Discussion

Many instances of blocking ELISAs have been previously described, especially those used to detect virus-specific antibodies in complex sera [21,22,23], whereby an ELISA plate is coated with a viral antigen, incubated with a titration series of test sera, and then probed with a specific detection mAb to generate a signal readout. In these examples, the presence of antigen-specific sera antibodies acts as the ‘blocker’ and decreases binding of the detection mAb and the corresponding ELISA signal. This differs from our blocking ELISA, where one mAb was intentionally selected to act as a blocker for another mAb to allow the detection of a specific protein variant in bivalent samples. To our knowledge, the epitope-blocking ELISA for the Beta spike antigen may be one of the few, if not the only, blocking ELISAs developed and transferred to QC laboratories to test industrial protein vaccine samples as an in vitro potency assay. A similar blocking ELISA strategy can be applied to other projects involving two highly related proteins to help overcome specific mAb availability issues that may exist.

Traditionally, vaccines have relied on animal-based potency tests that have proven to be costly and highly variable. As such, there is increased focus on animal-free potency assays, driven by the ‘3Rs’ initiatives (Replacement, Reduction, and Refinement of animal testing) [24]. Notably, there is more industry alignment with animal-free ‘consistency approaches’ for releasing vaccines whereby a vaccine batch must meet an expected ‘product profile’ based on relevant physicochemical and/or immunological test methods [25].

The identification of antibodies with desirable properties including functionality and the ability to detect changes in target epitope stability is key for the development of relevant in vitro potency assays for vaccines [9,10,11,26]. In the context of COVID-19 spike antigen-based vaccines, antibodies directed against the RBD are of particular interest since this region mediates binding of the virus to the host cells [27].

The emergence of new SARS-CoV-2 variants of concern posed challenges for vaccine developers, including those developing analytical methods such as antibody-based potency assays. Facing accelerated vaccine development and testing demands imparted by the COVID-19 pandemic, an epitope-blocking ELISA strategy using existing mAbs originally generated against only the ancestral spike antigen proved feasible for the specific detection of the Beta spike antigen in bivalent candidate vaccines containing both ancestral and Beta spike protein variants.

To achieve this, the rapid screening of candidate mAbs possessing the desired properties was crucial. BLI proved to be very versatile for this purpose as it was able to perform qualitative and quantitative binding kinetics, binding affinity, and epitope binning, which helped to identify the lead candidate ‘blocking’ mAb for one spike antigen and the subsequent detection mAb for the other variant antigen. While other biosensor platforms (e.g., SPR) could perform similar tasks, the simplicity of use of the BLI system, not requiring complicated liquid pumps, microfluidic flow cells, chip immobilization steps, or extensive cleaning, made it ideal to use in this situation. Furthermore, BLI was able to simulate the blocking ELISA steps as a ‘proof of concept’ for our assay. Others have similarly exploited BLI to assess ligand–target interactions and predict ELISA performance [28].

HDX-MS analysis furthered the understanding of the epitopes involved in the blocking ELISA. On the ancestral spike antigen, the epitope region bound by the detection mAb 876 used in the blocking ELISA was in close proximity (within 20 amino acids) to the epitope region bound by the blocking mAb 879. While these regions did not directly overlap, the binding of blocking mAb 879 to the ancestral spike antigen could conceivably result in steric hindrance, would interfere with the subsequent binding of the ELISA detection mAb to this spike variant, which was key to the success of the blocking ELISA detecting exclusively the Beta variant in bivalent formulations.

We note that when the blocking ELISA was performed without the mAb 879 ‘blocking step’ (hence essentially becoming an ELISA with mAb 875 capture and mAb 876 detection), the total antigenicity values measured in various bivalent formulations were lower than expected (see Table 2; the results in are in the orange column, compared to the expected total amount of all spike antigens shown in the first table column). In addition, the results for a monovalent formulation containing only an ancestral spike antigen were also notably lower than expected under such test conditions. The induction of allosteric effects on SARS-CoV-2 spike antigens by mAb binding events is well documented [29]; hence, it is conceivable that the binding of certain mAbs could lead to allosteric effects that might alter the accessibility of other mAb binding sites on certain spike antigen variants, resulting in lower-than-expected results from a sandwich ELISA. Our HDX-MS data supports this (Supplementary Figure S9), with evidence that the interaction of the ELISA capture mAb 875 resulted in spike antigen conformational changes in the S1 region. Upon complexations, there were regions in the S1 domain that showed notable changes, for example, residues 291–305 from the ancestral variant and their equivalents in the Beta variant (residues 288–309). Similarly, residues 556–570 and 624–681 of the ancestral variant and residues 553–569 and 611–620 of the Beta variant showed a decrease in deuterium exchange. While these regions do not map directly to the identified epitopes for any of the anti-S1 mAbs used in our ELISAs (Table 3), upon visualizing onto a 3D structure (Supplementary Figure S9), they correspond to NTD, CTD1, and CTD2 domains, which may influence RBD positioning [30]. It is possible that cumulative allosteric changes may have affected the binding of the subsequent detection mAb (and hence ELISA results) more on the ancestral spike antigen compared to the Beta variant; however, this would require further studies beyond the scope of this current report.

Controlling or reducing the effects of allostery (or other modifications such as protein glycosylation) that could impact an ELISA readout can be difficult. The use of reference standard spike antigen(s) obtained from the same expression system and purified with the same process as spike antigens used to formulate vaccine samples can help, as both should experience relatively similar allosteric effects in an ELISA. Ensuring consistent antigen purification processes would also better align post-translational modifications between the reference standard and different sample batches, thus reducing ELISA results variability as well, assuming such protein modifications might interfere with ELISA mAb binding. Regardless, it is evident that the blocking ELISA and the original developed ELISA are specific for the Beta and ancestral spike antigens, respectively, and typically measure expected values upon testing various formulations to demonstrate suitability for their intended purpose.

Following the method qualifications described in this report, the epitope-blocking ELISA for measuring Beta spike antigenicity in bivalent formulations, as well as the original ELISA specific for the ancestral spike antigen, were ultimately transferred to commercial industrial quality control (QC) laboratories, where they were validated to confirm accuracy, precision (intermediate precision and repeatability), linearity, and specificity for their respective spike antigen variants. Importantly, the epitope-blocking ELISA served as an interim testing solution for the QC laboratories to monitor the Beta spike antigen in bivalent COVID-19 developmental formulations. Eventually, mAbs generated by HuCAL specifically against the Beta spike antigen were acquired and used for further in vitro potency ELISA development without the need for a blocking step.

## 5. Conclusions

The current study demonstrates how the different binding properties of mAbs raised against one target protein can be exploited to develop an antibody-based assay specific to another closely related protein, especially in samples that contain both target variants. In particular, BLI proved to be an essential and rapid tool to help drive antibody characterization and decision making forward. HDX-MS also provided valuable insight into how the blocking ELISA strategy worked. This strategy proved useful to deliver an interim assay solution for a time-sensitive project, driven by increased vaccine testing demands and the lack of readily available specific mAbs for a new variant of concern during the COVID-19 pandemic.

## Figures and Tables

**Table 1:** Screening binding affinities, neutralizing titer, and ability to inhibit spike antigen interaction with ACE2 receptor amongst subset of monoclonal antibodies generated against purified ancestral SARS-CoV-2 spike antigen.

Ancestral Spike Antigen Domain	mAb Clone Number	Equilibrium Dissociation Constant; KD (M)	Association *k_on_* (1/Ms)	Dissociation *k_off_* (1/s)	Neutralizing Titer (ng/mL) ^1^	Reduction/Inhibition of Ancestral Spike Antigen Binding to ACE2 Receptor ^2^
S1	876	3.01 × 10^−10^	2.60 × 10^5^	7.83 × 10^−5^	76	+
S1	879	6.52 × 10^−10^	3.91 × 10^5^	2.55 × 10^−4^	93	+++
S1	886	5.39 × 10^−10^	2.00 × 10^5^	1.08 × 10^−4^	27	+++
S1	962	1.51 × 10^−9^	3.22 × 10^5^	4.87 × 10^−4^	6	+++
S2	875	9.29 × 10^−10^	4.19 × 10^5^	3.89 × 10^−4^	Not assessed	Not assessed

^1^ Determined using the pseudovirus neutralization assay. ^2^ Assessed using the BLI assay. The “+++” indicates 0% to 30%, and “+” indicates 61% to 90% of spike antigen binding signal to ACE2 remaining with the highest concentration of mAb used.

**Table 2:** Assessment of Beta and ancestral spike antigen antigenicity values obtained from various bivalent spike antigen formulations using epitope-blocking ELISA (with monovalent Beta spike antigen reference standard) and original ancestral spike antigen-specific ELISA (with monovalent ancestral spike antigen reference standard).

Bivalent Sample (Ancestral/Beta) (μg/mL)	Beta Spike Antigen Antigenicity Using Epitope-Blocking ELISA (ETU/mL) ^1^	% Recovery of Beta Spike Antigen with Epitope-Blocking ELISA ^2^	Antigenicity Results Using mAb 875 (Capture) and mAb 876 (Detection) Without any mAb 879 Blocking Step ^1, 3^	Ancestral Spike Antigen Antigenicity Using Originally Developed ELISA ^1^	% Recovery of Ancestral Spike Antigen Using Originally Developed ELISA ^2^
5.0/5.0	5.12	102%	5.96	4.55	91%
2.5/2.5	2.52	101%	3.1	2.13	85%
1.25/1.25	1.21	97%	1.47	1.02	82%
0.625/0.625	0.6	96%	0.71	0.53	85%
2.5/0	0	No recovery, as expected	0.63	2.3	92%
0/2.5	2.48	99%	2.34	0	No recovery, as expected
5.0/2.5	2.45	98%	3.54	4.95	99%

^1^ Antigenicity is reported as ELISA test units/mL (ETU/mL). ^2^ Percent recovery is expressed as antigenicity value (ETU/mL) divided by the expected formulated concentration (μg/mL) × 100. ^3^ Samples were tested using the epitope-blocking ELISA procedure and monovalent Beta spike antigen reference standard, but without the blocking mAb step.

**Table 3:** Epitope mapping of selected SARS-CoV-2 spike antigen mAbs using HDX-MS. Common sequences detected in both ancestral and Beta epitopes are underlined.

mAb Clone	mAb Role	Epitope (aa) on Ancestral Spike Antigen	Epitope (aa) on Beta Spike Antigen
Anti-S1			
876	Detection antibody for Beta spike antigen in blocking ELISA	420–439 (VIAWNSNNLDSKVGGNYNYL)	425–436 (LDSKVGGNYNYL)
879	Blocking antibody for ancestral spike antigen	459–475 (IYQAGSTPCNGVEGFNC)	No mAb binding (no notable HDX-MS difference observed; see Supplementary Figure S8)
886	Detection antibody for ancestral spike antigen	459–475 (IYQAGSTPCNGVEGFNC)	No data available
Anti-S2			
875	Capture mAb	771–781 (QVKQIYKTPPI) 856–872 (IAQYTSALLAGTITSG)	754–766 (IAVEQDKNTQEVF) 853–861 (MIAQYTSAL)

**Table 4:** Summary of intermediate precision and accuracy (percent recovery) of the epitope-blocking ELISA for the Beta spike antigen in bivalent formulations.

Sample (Level of Beta Spike Antigen in Bivalent Formulation)	Antigenicity (ETU/mL) ^1^	Average Antigenicity (ETU/mL)	%CV	Concentration of Beta Spike Antigen Used in Formulation (µg/mL)	Average % Recovery
Level 5 (200%)	41.4	40.2	2.5	40	101
	39.7				
	39.6				
Level 4 (100%)	21.3	20.5	6.1	20	103
	21.2				
	19.1				
Level 3 (50%)	10.6	10.0	5.6	10	100
	9.9				
	9.5				
Level 2 (25%)	4.9	4.8	5.5	5	96
	5.0				
	4.5				
Level 1 (12.5%)	2.7	2.5	8.4	2.5	99
	2.3				
	2.4				

^1^ Monovalent Beta spike antigen used as a reference standard.

## Data Availability

ELISA development, BLI, and HDX-MS raw data can be provided if requested and if cleared for sharing by Sanofi Intellectual Property and/or Legal departments.

## Supplementary Materials

The following supporting information can be downloaded at: https://www.mdpi.com/article/10.3390/vaccines13080794/s1, Figure S1: Amino acid sequence comparison between the SARS-CoV-2 spike antigen for both the recombinant ancestral and Beta variants. Modification to the furin cleavage site is highlighted in yellow, prolines to stabilize pre-fusion conformation are highlighted in green, and the C-terminal T4 bacteriophage fibritin foldon domain are bolded and underlined; Figure S2: (A-D) Using BLI to assess inhibition/reduction of spike antigen interaction with ACE2 receptor by anti-S1 domain mAbs; Figure S3: Example of dot blot analyses of various mAbs to determine whether they bind to conformational or denatured/linear ancestral spike antigen epitopes; Figure S4: 4-PL response curves for ancestral spike antigen ELISA using 16-point dilutions; Figure S5: BLI experiment confirming that mAb 879 is unable to block detection of ancestral spike antigen by mAb 962; Figure S6: BLI shows mAb 876 is able to detect changes in heat stressed Beta spike antigen previously treated for 4 days at 37 °C, 45 °C, and 60 °C as indicated; Figure S7: Example of 4-PL response curves for the epitope blocking ELISA for Beta spike antigen antigenicity using 8-point dilutions and anchoring of curves to the median optical density value of ELISA blank wells. Red response curve shows reference standard (monovalent Beta spike antigen lot in this case) and blue curve shows a bivalent formulation sample (containing ancestral and Beta spike antigens; Figure S8: HDX-MS difference plots for mAb 876/ancestral spike antigen, mAb 876/Beta spike antigen, mAb879/ancestral spike antigen, mAb879/ Beta spike antigen, and mAb 886/ancestral spike antigen complexes; Figure S9: HDX-MS difference plots for mAb 875/ancestral spike antigen and mAb 875/Beta spike antigen complexes; Figure S10. Linearity assessment of epitope blocking ELISA for Beta spike antigen antigenicity from five concentration levels of bivalent ancestral/Beta spike antigen formulations. Results obtained using monovalent Beta spike antigen as a reference standard; Figure S11: Linearity assessment of ELISA for ancestral spike antigenicity from five concentration levels of bivalent ancestral/Beta spike antigen formulations. Results obtained using monovalent ancestral spike antigen as a reference standard; Table S1: Screening binding affinities, neutralizing titer, and ability to inhibit spike antigen interaction with ACE2 receptor amongst monoclonal antibodies generated against purified ancestral SARS-CoV-2 spike antigen; Table S2: Binding kinetics for selected monoclonal antibodies to purified ancestral and Beta spike antigens; Table S3: Assessment of Beta spike antigen antigenicity values obtained from various bivalent ancestral/Beta spike antigen formulations using epitope blocking ELISA and bivalent reference standard; Table S4: Bivalent ancestral/Beta spike antigen formulations used for assay qualifications; Table S5: Summary of intermediate precision and accuracy (percent recovery) obtained from bivalent formulations using the original ELISA developed for ancestral spike antigen.
