# Supplementary material for: Epitope Profiling of SARS-CoV-2 Spike Antigen Provides a Novel Strategy for Developing ELISAs Specific for Different Spike Protein Variants in Bivalent Vaccine Formulations
Source: Vaccines (Basel). 2025 Jul 26;13(8):794. doi: 10.3390/vaccines13080794 (PMC12390492; doi:10.3390/vaccines13080794)
Supplement: Supplementary file 1 [file vaccines-13-00794-s001.zip › vaccines-3645259-supplementary.pdf]

Supplementary Figures

```
Ancestral       QCVNLTTRTQLPPAYTNSFTRGVYYPDKVFRSSVLHSTQDLFLPFFSNVTWFHAIHVSGT     60
Beta            QCVNFTTRTQLPPAYTNSFTRGVYYPDKVFRSSVLHSTQDLFLPFFSNVTWFHAIHVSGT     60
                ****:*******************************************************

Ancestral       NGTKRFDNPVLPFNDGVYFASTEKSNIIRGWIFGTTLDSKTQSLLIVNNATNVVIKVCEF     120
Beta            NGTKRFANPVLPFNDGVYFASTEKSNIIRGWIFGTTLDSKTQSLLIVNNATNVVIKVCEF     120
                ****** *****************************************************

Ancestral       QFCNDPFLGVYYHKNNKSWMESEFRVYSSANNCTFEYVSQPFLMDLEGKQGNFKNLREFV     180
Beta            QFCNDPFLGVYYHKNNKSWMESEFRVYSSANNCTFEYVSQPFLMDLEGKQGNFKNLREFV     180
                ************************************************************

Ancestral       FKNIDGYFKIYSKHTPINLVRDLPQGFSALEPLVDLPIGINITRFQTLLALHRSYLTPGD     240
Beta            FKNIDGYFKIYSKHTPINLVRGLPQGFSALEPLVDLPIGINITRFQT---LHRSYLTPGD     237
                *********************.*************************   **********

Ancestral       SSSGWTAGAAAYYVGYLQPRTFLLKYNENGTITDAVDCALDPLSETKCTLKSFTVEKGIY     300
Beta            SSSGWTAGAAAYYVGYLQPRTFLLKYNENGTITDAVDCALDPLSETKCTLKSFTVEKGIY     297
                ************************************************************

Ancestral       QTSNFRVQPTESIVRFPNITNLCPFGEVFNATRFASVYAWNRKRISNCVADYSVLYNSAS     360
Beta            QTSNFRVQPTESIVRFPNITNLCPFGEVFNATRFASVYAWNRKRISNCVADYSVLYNSAS     357
                ************************************************************

Ancestral       FSTFKCYGVSPTKLNDLCFTNVYADSFVIRGDEVRQIAPGQTGKIADYNYKLPDDFTGCV     420
Beta            FSTFKCYGVSPTKLNDLCFTNVYADSFVIRGDEVRQIAPGQTGNIADYNYKLPDDFTGCV     417
                *******************************************:****************

Ancestral       IAWNSNNLDSKVGGNYNYLYRLFRKSNLKPFERDISTEIYQAGSTPCNGVEGFNCYFPLQ     480
Beta            IAWNSNNLDSKVGGNYNYLYRLFRKSNLKPFERDISTEIYQAGSTPCNGVKGFNCYFPLQ     477
                **************************************************:*********

Ancestral       SYGFQPTNGVGYQPYRVVVLSFELLHAPATVCGPKKSTNLVKNKCVNFNFNGLTGTGVLT     540
Beta            SYGFQPTYGVGYQPYRVVVLSFELLHAPATVCGPKKSTNLVKNKCVNFNFNGLTGTGVLT     537
                ******* ****************************************************

Ancestral       ESNKKFLPFQQFGRDIADTTDAVRDPQTLEILDITPCSFGGVSVITPGTNTSNQVAVLYQ     600
Beta            ESNKKFLPFQQFGRDIADTTDAVRDPQTLEILDITPCSFGGVSVITPGTNTSNQVAVLYQ     597
                ************************************************************

Ancestral       DVNCTEVPVAIHADQLTPTWRVYSTGSNVFQTRAGCLIGAEHVNNSYECDIPIGAGICAS     660
Beta            GVNCTEVPVAIHADQLTPTWRVYSTGSNVFQTRAGCLIGAEHVNNSYECDIPIGAGICAS     657
                .***********************************************************

Ancestral       YQTQTNSPGSASSVASQSIIAYTMSLGAENSVAYSNNSIAIPTNFTISVTTEILPVSMTK     720
Beta            YQTQTNSPGSASSVASQSIIAYTMSLGVENSVAYSNNSIAIPTNFTISVTTEILPVSMTK     717
                ***************************.********************************

Ancestral       TSVDCTMYICGDSTECSNLLLQYGSFCTQLNRALTGIAVEQDKNTQEVFAQVKQIYKTPP     780
Beta            TSVDCTMYICGDSTECSNLLLQYGSFCTQLNRALTGIAVEQDKNTQEVFAQVKQIYKTPP     777
                ************************************************************

Ancestral       IKDFGGFNFSQILPDPSKPSKRSFIEDLLFNKVTLADAGFIKQYGDCLGDIAARDLICAQ     840
Beta            IKDFGGFNFSQILPDPSKPSKRSFIEDLLFNKVTLADAGFIKQYGDCLGDIAARDLICAQ     837
                ************************************************************

Ancestral       KFNGLTVLPPLLTDEMIAQYTSALLAGTITSGWTFGAGAALQIPFAMQMAYRFNGIGVTQ     900
Beta            KFNGLTVLPPLLTDEMIAQYTSALLAGTITSGWTFGAGAALQIPFAMQMAYRFNGIGVTQ     897
                ************************************************************

Ancestral       NVLYENQKLIANQFNSAIGKIQDSLSSTASALGKLQDVVNQNAQALNTLVKQLSSNFGAI     960
Beta            NVLYENQKLIANQFNSAIGKIQDSLSSTASALGKLQDVVNQNAQALNTLVKQLSSNFGAI     957
                ************************************************************

Ancestral       SSVLNDILSRLDPPEAEVQIDRLITGRLQSLQTYVTQQLIRAAEIRASANLAATKMSECV     1020
Beta            SSVLNDILSRLDPPEAEVQIDRLITGRLQSLQTYVTQQLIRAAEIRASANLAATKMSECV     1017
                ************************************************************

Ancestral       LGQSKRVDFCGKGYHLMSFPQSAPHGVVFLHVTYVPAQEKNFTTAPAICHDGKAHFPREG     1080
Beta            LGQSKRVDFCGKGYHLMSFPQSAPHGVVFLHVTYVPAQEKNFTTAPAICHDGKAHFPREG     1077
                ************************************************************

Ancestral       VFVSNGTHWFVTQRNFYEPQIITTDNTFVSGNCDVVIGIVNNTVYDPLQPELDSFKEELD     1140
Beta            VFVSNGTHWFVTQRNFYEPQIITTDNTFVSGNCDVVIGIVNNTVYDPLQPELDSFKEELD     1137
                ************************************************************
```

```
Ancestral      KYFKNHTSPDVDLGDISGINASVVNIQKEIDRLNEVAKNLNESLIDLQELGKYEQYIKGY      1200
Beta           KYFKNHTSPDVDLGDISGINASVVNIQKEIDRLNEVAKNLNESLIDLQELGKYEQYIKGY      1197
               ************************************************************

Ancestral      IPEAPRDGQAYVRKDGEWVFLSTFL        1225
Beta           IPEAPRDGQAYVRKDGEWVFLSTFL        1222
               *************************
```

Supplementary Figure S1: Amino acid sequence comparison between the SARS-CoV-2 spike antigen for both the recombinant ancestral and Beta variants. Modification to the furin cleavage site is highlighted in yellow, prolines to stabilize pre-fusion conformation are highlighted in green, and the C-terminal T4 bacteriophage fibritin foldon domain are bolded and underlined.

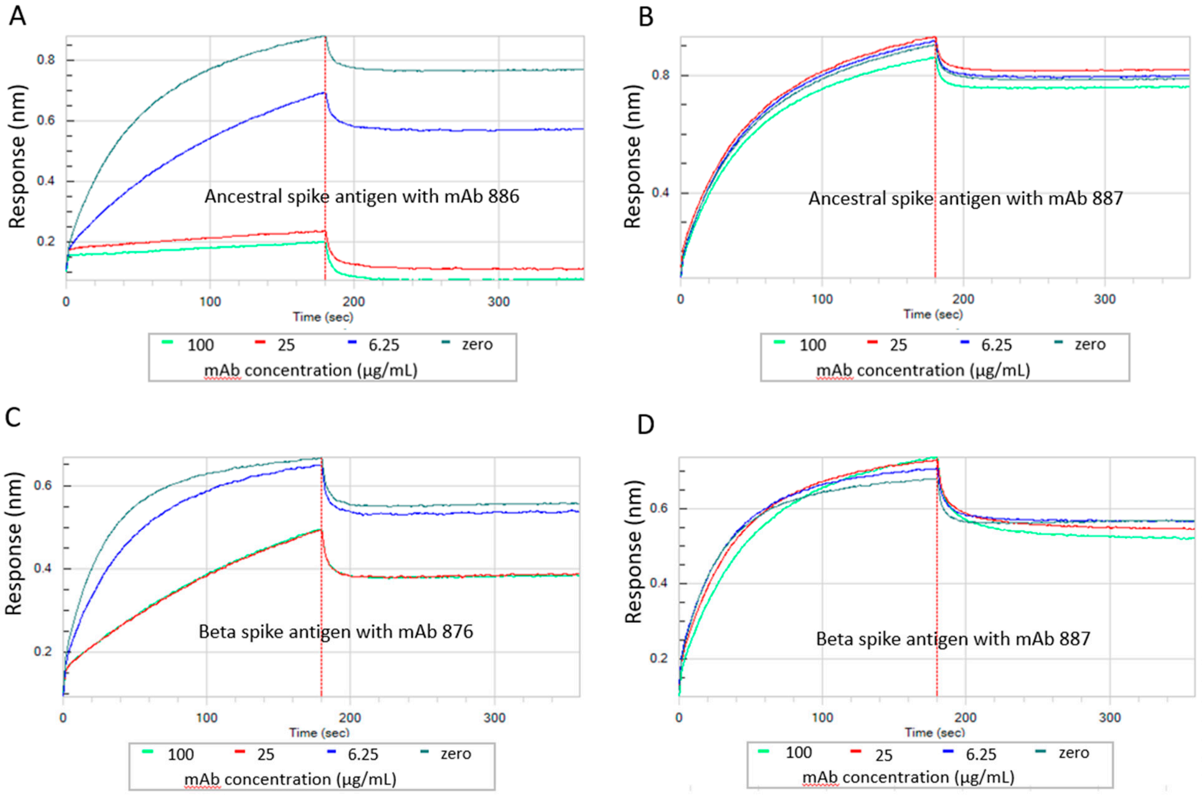

Supplementary Figure S2: (A-D) Using BLI to assess inhibition/reduction of spike antigen interaction with ACE2 receptor by anti-S1 domain mAbs. Panel (A) shows example of concentration dependent ability of mAb 886 to inhibit the interaction of ancestral spike antigen with ACE2 receptor captured on BLI sensor, while (B) shows mAb 887 was not able to inhibit the interaction at any of the antibody concentrations tested. Panel (C) shows example of mAb 876 impairing the interaction of Beta spike antigen with ACE2 receptor, while (D) shows mAb 887 was not able to inhibit the interaction.

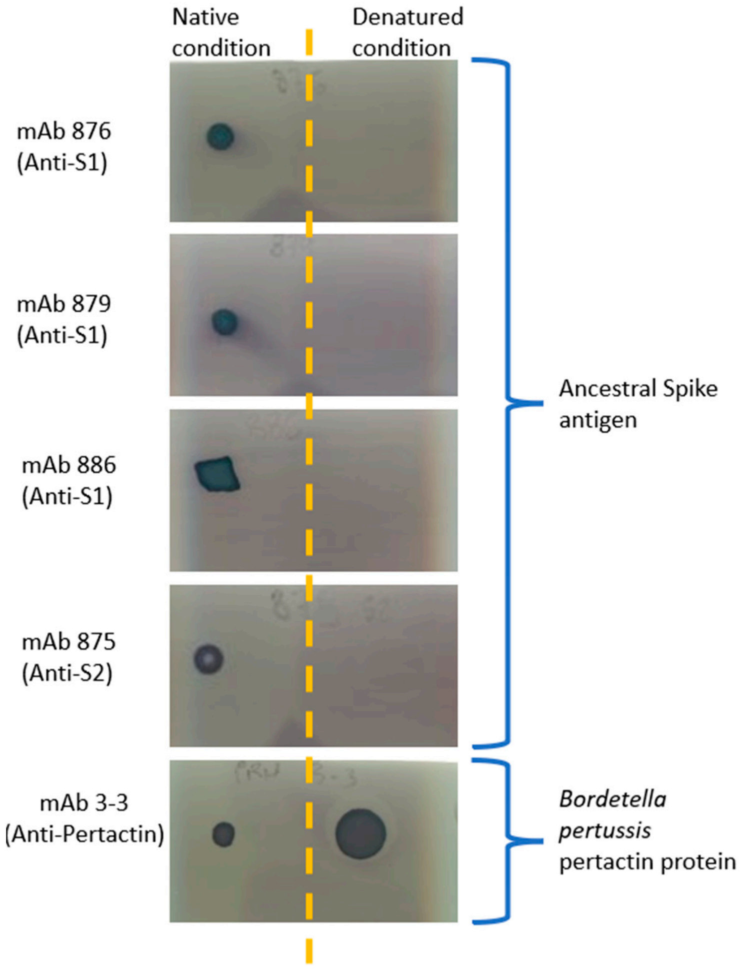

Supplementary Figure S3: Example of dot blot analyses of various mAbs to determine whether they bind to conformational or denatured/linear ancestral spike antigen epitopes. PVDF membranes were spotted with purified native ancestral spike antigen (prepared in Dulbecco's phosphate-buffered saline (DPBS buffer) or denatured ancestral spike antigen (prepared in DPBS buffer, 0.1% Rapigest (Waters Corporation), and 1X reducing agent (Novex), and heat treated at 95°C for 10 min). After blocking, the membranes were probed with each mAb (1 µg/mL) and immunodetection was done using WesternBreeze Immunodetection kit (Novex). An unrelated antibody and protein control (mAb 3-3 raised against *Bordetella pertussis* pertactin antigen) was included to demonstrate a mAb that can bind to a linear (denatured) protein target.

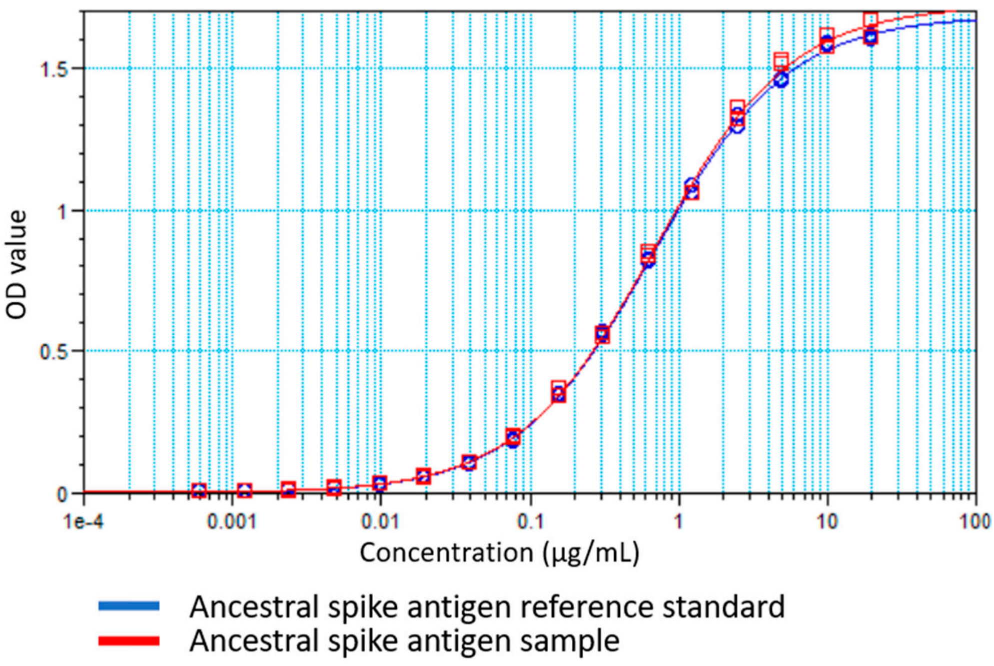

Supplementary Figure S4: 4-PL response curves for ancestral spike antigen ELISA using 16-point dilutions.

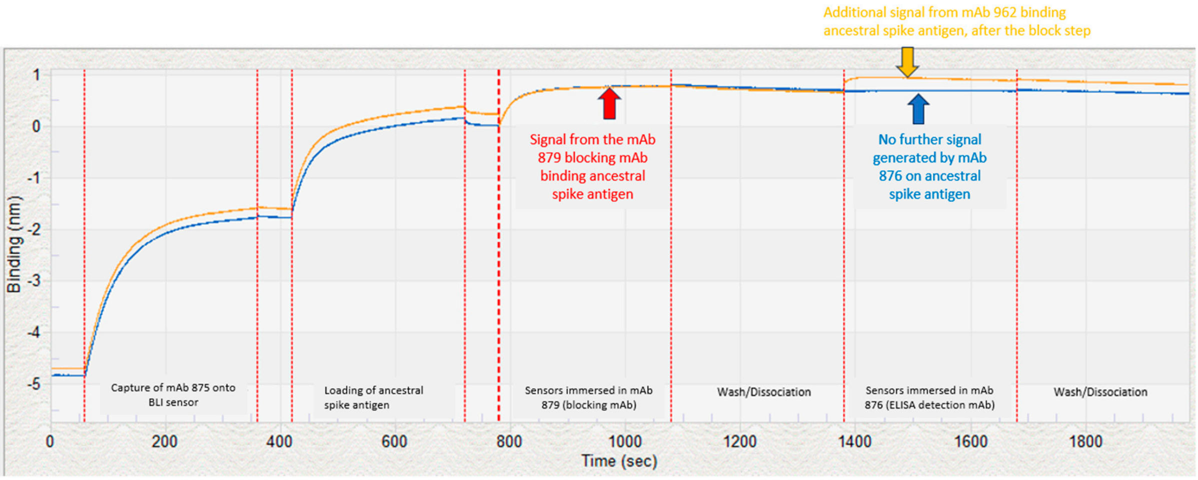

Supplementary Figure S5: BLI experiment confirming that mAb 879 is unable to block detection of ancestral spike antigen by mAb 962. The mAb 875 (used as the capture antibody in the blocking ELISA) was first immobilized on BLI biosensors. Sensors were then exposed to ancestral spike antigen to allow binding. Sensors were then placed into solution of the blocking mAb candidate (mAb 879, see step indicated with red arrow), washed, and then exposed to mAb 962 or mAb 876. Sensors that were immersed into mAb 962 were still able to generate a signal, indicating the mAb 962 epitope was not blocked (orange arrow). In contrast, sensors exposed to mAb 876 did not generate any further signal (blue arrow), indicating the mAb 876 epitope was no longer available to bind.

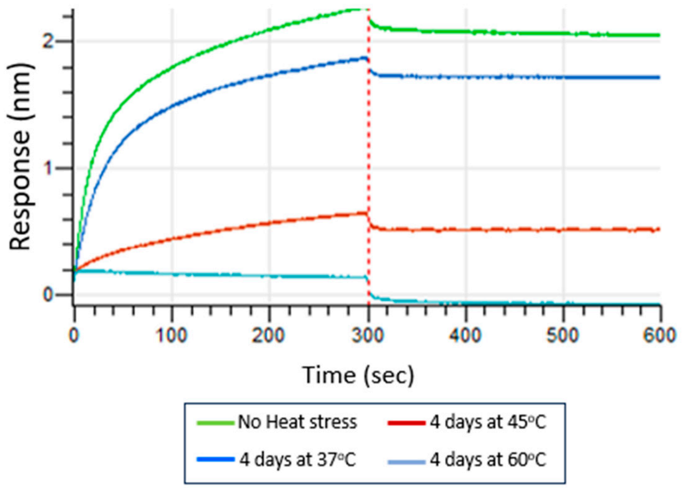

Supplementary Figure S6: BLI shows mAb 876 is able to detect changes in heat stressed Beta spike antigen previously treated for 4 days at 37ºC, 45ºC, and 60ºC as indicated.

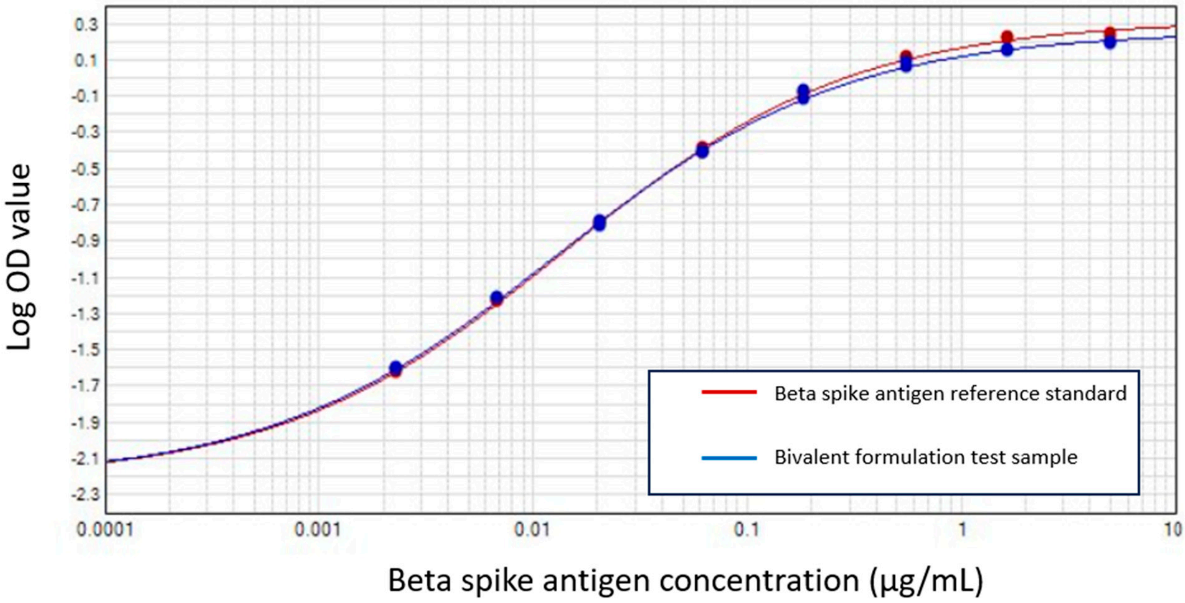

Supplementary Figure S7. Example of 4-PL response curves for the epitope blocking ELISA for Beta spike antigen antigenicity using 8-point dilutions and anchoring of curves to the median optical density value of ELISA blank wells. Red response curve shows reference standard (monovalent Beta spike antigen lot in this case) and blue curve shows a bivalent formulation sample (containing ancestral and Beta spike antigens

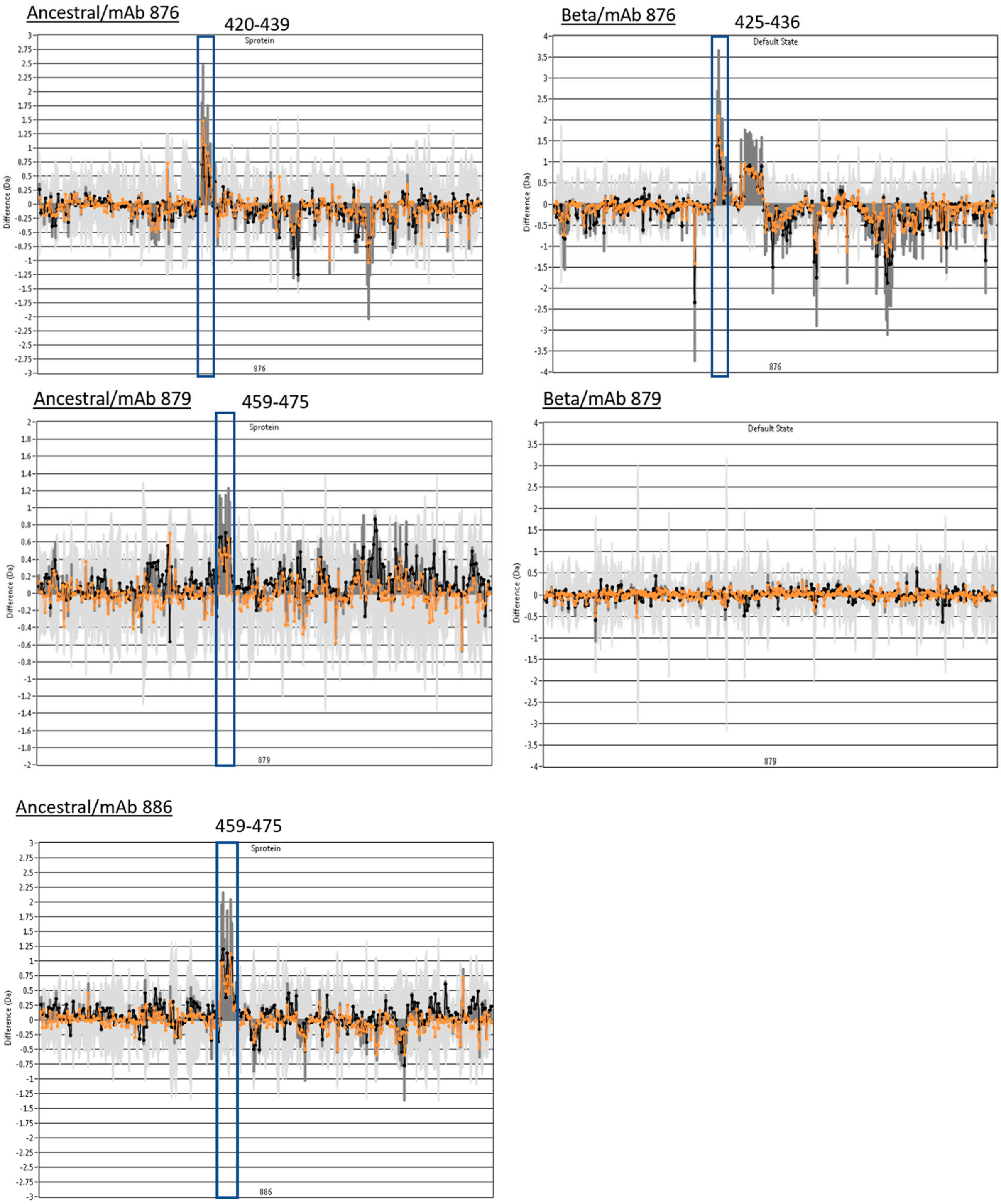

Supplementary Figure S8: HDX-MS difference plots for mAb 876/ancestral spike antigen, mAb 876/Beta spike antigen, mAb879/ancestral spike antigen, mAb879/ Beta spike antigen, and mAb 886/ancestral spike antigen complexes. Peptides from N- to C-terminus are shown on the x-axis from left to right respectively. Difference in deuterium uptake upon complexation is shown on the y-axis (decreased and increased uptake upon complexation is shown in positive and negative axis, respectively).

Grey shaded region represents 3x standard deviation. Ancestral spike antigen epitopes for mAb 876, 879, and 886 were 420-439 and 459-475 (for both 879 and 886 complexes), respectively. The Beta spike antigen epitope for mAb 876 is identified as residues 425-436. There was another region, residues 465-493, that also surpassed the statistically significant criteria. However, this could be due to steric hindrance effect upon binding. No major differences were observed for mAb 879/Beta complex supporting no interaction between the two.

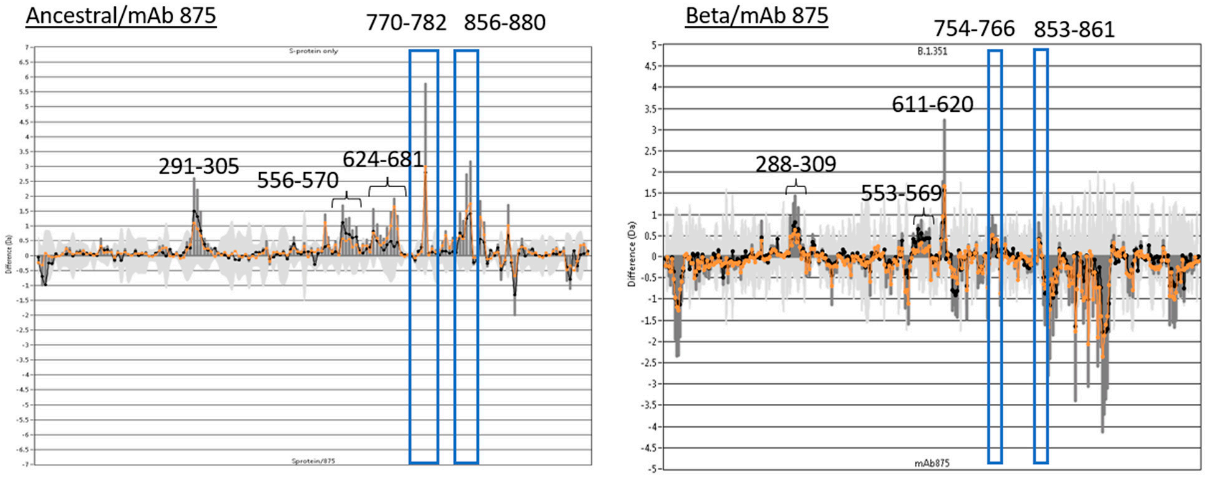

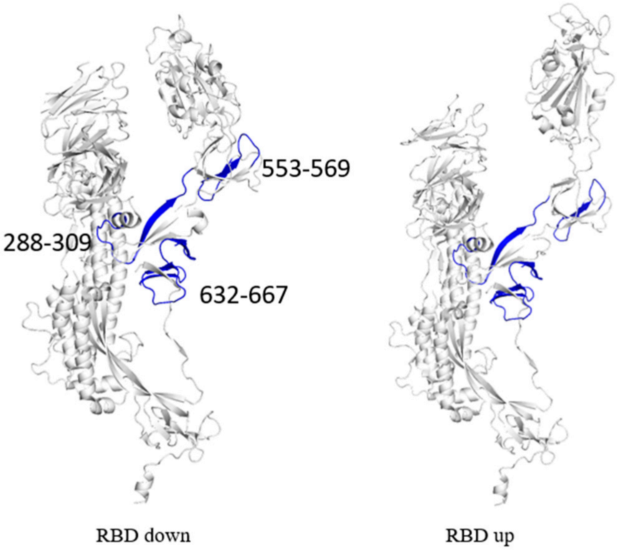

Supplementary Figure S9: Top **-** HDX-MS difference plots for mAb 875/ancestral spike antigen and mAb 875/Beta spike antigen complexes. Peptides from N- to C-terminus are shown on the x-axis from left to right respectively. Difference in deuterium uptake upon complexation is shown on the y-axis (decreased and increased uptake upon complexation is shown in positive and negative axis, respectively). Grey shaded region represents 3x standard deviation. In both variants, upon complexations, there were regions in S1 domain that showed statistically significant changes. For example, residues 291-305 from ancestral and its equivalent in Beta variant (288-309). Similarly, residues 556-570 and 624-681 for Ancestral variant and residues 553-569 and 611-620 for Beta variant showed drastic decrease as well. Upon visualizing onto 3D structure (bottom), these regions correspond to NTD, CTD1, and CTD2 domains which may influence RBD positioning (only residues 288-309, 553-569, and 632-667 were mapped as the other regions were missing in the crystal structure, PDB: 7LYQ). As such, mAb 875 binding in the S2 domain seemed to have caused allosteric effect in these regions. There were two common regions in S2 domain, and the epitope has been assigned at residues 770-782 and 856-880 for ancestral spike antigen and at residues 754-766 and 853-861 for Beta spike antigen. The refined epitope

for ancestral spike antigen were residues 771-781 and 856-872 as the neighboring peptides showed negligible deuteration differences.

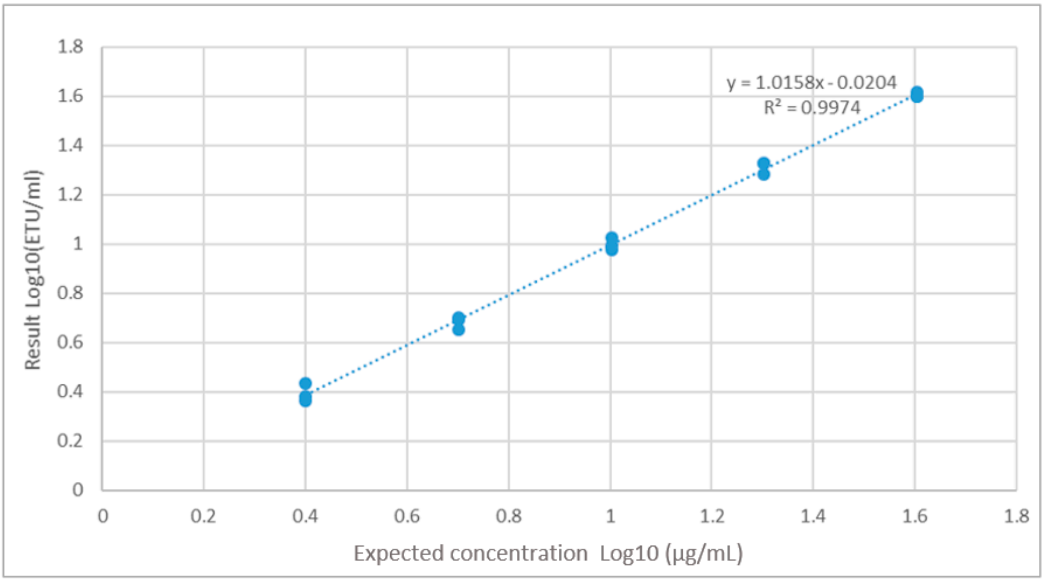

Supplementary Figure S10. Linearity assessment of epitope blocking ELISA for Beta spike antigen antigenicity from five concentration levels of bivalent ancestral/Beta spike antigen formulations. Results obtained using monovalent Beta spike antigen as a reference standard.

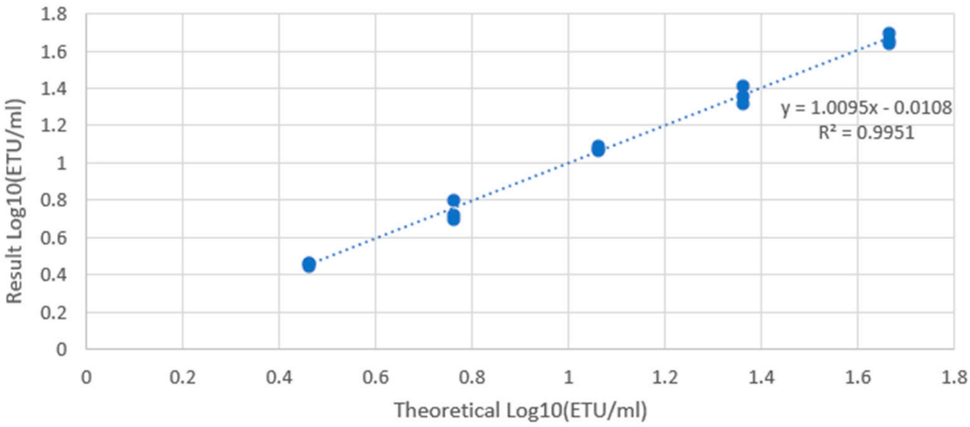

Supplementary Figure S11: Linearity assessment of ELISA for ancestral spike antigenicity from five concentration levels of bivalent ancestral/Beta spike antigen formulations. Results obtained using monovalent ancestral spike antigen as a reference standard.

Supplementary Tables

Supplementary Table S1: Screening binding affinities, neutralizing titer, and ability to inhibit spike antigen interaction with ACE2 receptor amongst monoclonal antibodies generated against purified ancestral SARS-CoV-2 spike antigen.

Ancestral spike antigen targeted domain	mAb clone number	Equilibrium dissociation constant; KD (M)	Association k_{on} (1/Ms)	Dissociation k_{off} (1/s)	Neutralizing titer (ng/mL) [1]	Reduction/ Inhibition of ancestral spike antigen binding to ACE2 receptor [3]
S1	876	3.01×10^{-10}	2.60×10^{5}	7.83×10^{-5}	76	+
S1	877	3.01×10^{-10}	2.51×10^{5}	7.56×10^{-5}	833	-
S1	879	6.52×10^{-10}	3.91×10^{5}	2.55×10^{-4}	93	+++
S1	880	6.00×10^{-10}	4.84×10^{5}	2.90×10^{-4}	9,259	+
S1	881	1.45×10^{-9}	8.95×10^{5}	1.30×10^{-3}	625	-
S1	882	1.28×10^{-9}	4.07×10^{5}	5.19×10^{-4}	35,714	+
S1	883	1.00×10^{-9}	7.50×10^{5}	7.50×10^{-4}	833	+++
S1	884	3.89×10^{-10}	2.81×10^{5}	1.09×10^{-4}	250	+
S1	885	3.25×10^{-8}	7.13×10^{8}	Not determined [2]	125,000	+
S1	886	5.39×10^{-10}	2.00×10^{5}	1.08×10^{-4}	27	+++
S1	887	1.66×10^{-9}	4.36×10^{9}	Not determined [2]	>250,000	-
S1	962	1.51×10^{-9}	3.22×10^{5}	4.87×10^{-4}	6	+++
S1	963	8.27×10^{-10}	2.91×10^{5}	2.41×10^{-4}	179	+
S2	874	1.17×10^{-9}	4.96×10^{5}	5.78×10^{-4}	Not assessed	Not assessed
S2	875	9.29×10^{-10}	4.19×10^{5}	3.89×10^{-4}	Not assessed	Not assessed

[1] Determined using pseudovirus neutralization assay. [2] Dissociation rate not determined as mAb had poor binding characteristics, and poor alignment to kinetics curve fit models. [3] Assessed using BLI assay. The "-" indicates 90% or more of the spike antigen binding signal to ACE2 receptor was maintained even with the highest concentration of mAb used. The "+++" indicates 0% to 30%, "++" indicates 31% to 60%, and "+" indicates 61% to 90% of spike antigen binding signal to ACE2 remained with the highest concentration of mAb used.

Supplementary Table S2: Binding kinetics for selected monoclonal antibodies to purified ancestral and Beta spike antigens

Sample	mAb tested	Equilibrium dissociation constant (KD) (M)	Association k_{on} (1/Ms)	Dissociation k_{off} (1/s)
Beta spike antigen	mAb 886	3.09×10^{-9}	1.06×10^{6}	3.27×10^{-3}
	mAb 876	5.95×10^{-11}	1.92×10^{6}	1.14×10^{-4}
	mAb 879	1.93×10^{-8}	3.23×10^{5}	6.25×10^{-3}
Ancestral spike antigen	mAb 886	$<1.0 \times 10^{-12}$	2.52×10^{5}	$<1.0 \times 10^{-7}$
	mAb 876	$<1.0 \times 10^{-12}$	3.20×10^{5}	$<1.0 \times 10^{-7}$
	mAb 879	2.70×10^{-11}	3.68×10^{5}	9.94×10^{-6}

The binding kinetics of mAbs 886, 876, and 879 to Beta and ancestral spike antigen variants were formally assessed with BLI (using a concentration series of spike antigen concentrations).

Supplemental Table S3: Assessment of Beta spike antigen antigenicity values obtained from various bivalent ancestral/Beta spike antigen formulations using epitope blocking ELISA and bivalent reference standard.

Bivalent sample (µg/mL) (Ancestral /Beta)	Beta spike antigen Antigenicity using Epitope Blocking ELISA (ETU/mL) [1, 2] and Percent Recovery [3]			Average Antigenicity (ETU/mL) and Average Percent Recovery	%CV of Antigenicity results
	Run 1, Analyst 1 (Day 1)	Run 2, Analyst 2 (Day 2)	Run 3, Analyst 3 (Day 3)		
0/2.5	2.54 (101%)	2.90 (117%)	2.51 (101%)	2.63 (105%)	8%
2.5/5.0	5.76 (115%)	5.02 (100%)	5.22 (104%)	5.33 (107%)	7%
2.5/2.5	2.57 (103%)	2.92 (117%)	2.51 (101%)	2.67 (107%)	8%
2.5/1.25	1.30 (104%)	1.39 (111%)	1.43 (114%)	1.37 (110%)	5%
2.5/0.625	0.63 (101%)	0.63 (101%)	0.79 (119%)	0.67 (107%)	10%
2.5/0	No recovery, as expected	No recovery, as expected	No recovery, as expected	No recovery, as expected	No recovery, as expected

[1] Results obtained using bivalent formulation as a reference standard in this example. [2] Antigenicity reported as ELISA Test Units/ml (ETU/mL). [3] Percent recovery expressed as antigenicity value (ETU/mL) divided by the expected formulated concentration (µg/mL) x 100.

Supplementary Table S4: Bivalent ancestral/Beta spike antigen formulations used for assay qualifications.

Concentration Level	Final ancestral spike antigen concentration (µg/ml)	Final Beta spike antigen concentration (µg/ml)
**L5 (200%)**	40	40
**L4 (100%)**	20	20
**L3 (50%)**	10	10
**L2 (25%)**	5	5
**L1 (12.5%)**	2.5	2.5

Supplementary Table S5: Summary of intermediate precision and accuracy (percent recovery) obtained from bivalent formulations using the original ELISA developed for ancestral spike antigen [1]

**Sample (Level of ancestral spike antigen in bivalent formulation)**	**Antigenicity (ETU/ml)**	**Average Antigenicity (ETU/ml)**	**%CV**	**Concentration of ancestral spike antigen in formulation (µg/ml)**	**Average % Recovery**
**Level 5 (200%)**	45.6	46.3	5.6 %	40	116%
	43.6				
	49.8				
**Level 4 (100%)**	21.0	23.2	8.8 %	20	116%
	25.9				
	22.7				
**Level 3 (50%)**	11.7	12.0	2.1 %	10	120%
	11.9				
	12.3				
**Level 2 (25%)**	5.0	5.5	10.0 %	5	111%
	6.3				
	5.3				
**Level 1 (12.5%)**	2.8	2.9	1.6 %	2.5	115%
	2.9				
	2.9				

[1] Monovalent ancestral spike antigen used as reference standard.
